# Supplementary material for: A Global Screen for Assembly State Changes of the Mitotic Proteome by SEC-SWATH-MS
Source: Cell Syst. 2020 Feb 26;10(2):133–155.e6. doi: 10.1016/j.cels.2020.01.001 (PMC7042714; doi:10.1016/j.cels.2020.01.001)

Q15758 | AAAT_HUMAN | SLC1A5 ASCT2 M7V1 RDR RDRC
Monomer MW [kDa]: 56.598 Monomer expected elution fraction: 45

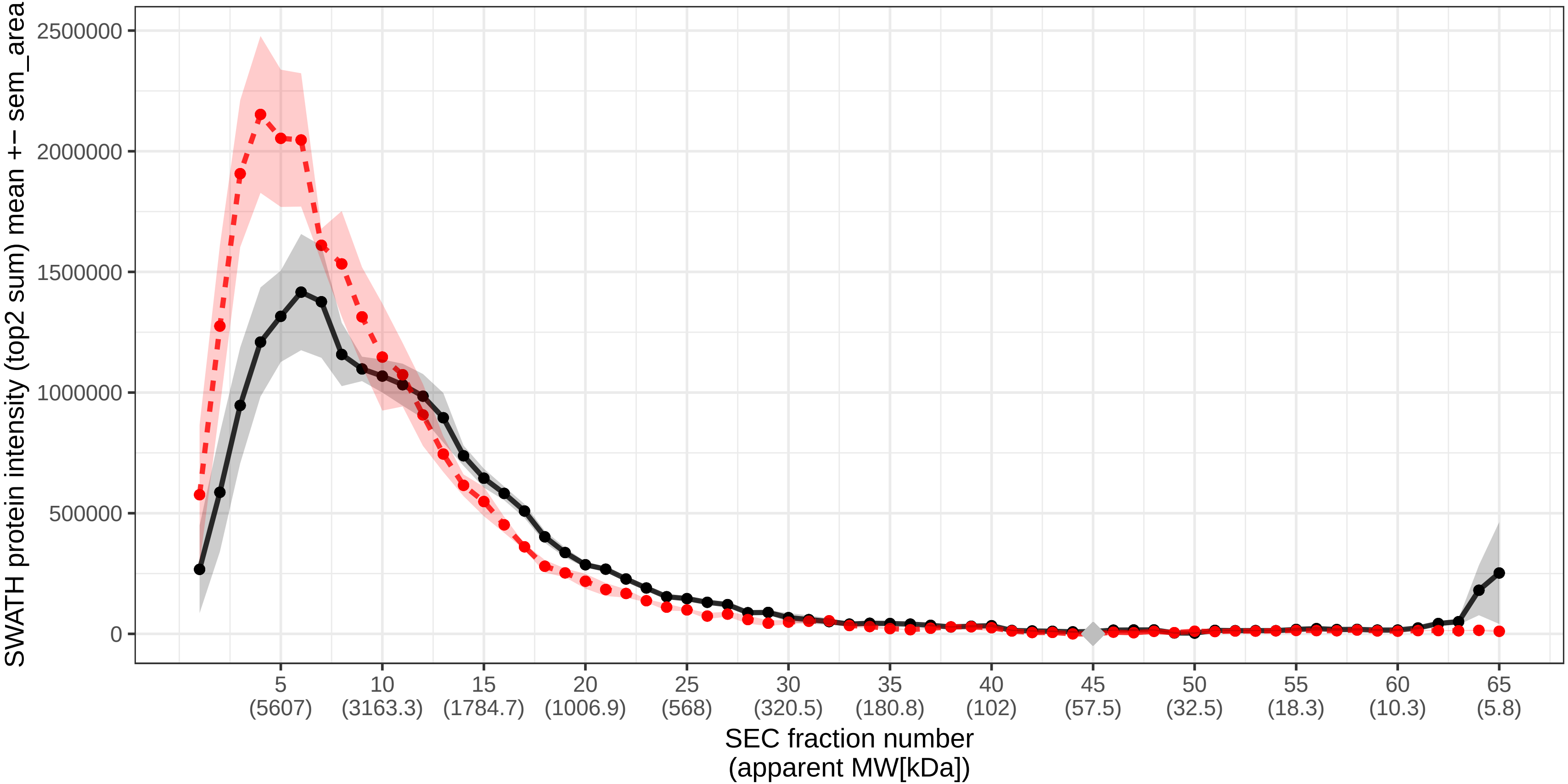

Supplement: Data S1. SEC-SWATH-MS Protein Chromatograms, Related to Figure 1 [file mmc6.zip › SECchrom_Q15758_AAAT_HUMAN_SLC1A5_ASCT2_M7V1_RDR_RDRC.pdf]
